# ‘Living in a shrinking world’—The experience of loneliness among community-dwelling older people with reduced mobility: a qualitative grounded theory approach

**DOI:** 10.1186/s12877-022-02998-5

**Published:** 2022-04-06

**Authors:** Marika Moeyersons, Kristel De Vliegher, Brooke Huyghe, Sacha De Groof, Koen Milisen, Bernadette Dierckx de Casterlé

**Affiliations:** 1 Department of Public Health and Primary Care, Academic Centre for Nursing and Midwifery, KU Leuven, Kapucijnenvoer 35 blok d bus, 7001, 3000 Leuven, Belgium; 2 Nursing Departement, Wit-Gele Kruis van Vlaanderen, Frontispiesstraat 8 bus 1.2 1000, Brussels, Belgium; 3 Department of Geriatric Medicine, University Hospital Leuven, Herestraat 49, 3000 Leuven, Belgium

**Keywords:** Loneliness, Mobility limitation, Community-dwelling older persons, Qualitative research

## Abstract

**Background:**

Loneliness is associated with severe consequences for both the physical and mental health of older people. Research has shown that mobility limitations are an important risk factor for the emergence and maintenance of feelings of loneliness. The aim of this study was to explore the experiences of loneliness among community-dwelling older people with reduced mobility and its underlying dynamics.

**Methods:**

This study employed a qualitative, descriptive design, inspired by a grounded theory approach. Participants were purposively recruited in collaboration with home health care providers. The main inclusion criteria were as follows: aged 75 years or older, being mobile impaired, experiencing feelings of loneliness, and living at home and being cognitively able to be interviewed. Data analysis of 15 semi-structured, in-depth interviews was conducted based on the Qualitative Analysis Guide of Leuven (QUAGOL).

**Results:**

Loneliness was expressed through all the stories, but it appeared as an elusive, intangible phenomenon. Both indicating the presence of loneliness and describing what this phenomenon means were difficult to express for most participants. Loneliness was experienced as an inherent part of the ageing process characterised by losses, limitations and meaninglessness. Participants described how they have experienced losing grip on the world in which they live and feel isolated in a literally and figuratively shrinking world. Loneliness is described as the feeling that one is unable to address the situation that results in deep sadness and the feeling of no longer being of value to their environment.

**Conclusions:**

This study shows that loneliness among community-dwelling older persons with reduced mobility is embedded in experiences of loss related to ageing, among which reduced mobility plays a significant role. The results suggest the existence of a more profound experience of loneliness than might appear at first glance. How to recognise experiences of loneliness and how to support a meaningful existence for community-dwelling older persons should be given priority in health care. The findings of this study can increase professional caregivers’ sensitivity to implicit signals of loneliness. Further research is necessary to refine the outcomes and to further explore the role of reduced mobility in the experience of loneliness.

**Supplementary Information:**

The online version contains supplementary material available at 10.1186/s12877-022-02998-5.

## Background

During the process of ageing, older people are exposed to a multitude of challenges and losses [1–3]. Changes occur in their social relationships due to the loss of important persons and meaningful contacts in their lives [4, 5]. They are faced with health problems, disability, and mortality [1, 2]. Their level of independence and mobility decrease over time [3, 6]. These factors contribute to the vulnerability of the older population and specifically to the risk of being affected by loneliness [3].

Although prevalence estimates vary across studies, the literature strongly suggests that old age increases the chance of feelings of loneliness [7–9]. Some studies have reported that the prevalence of loneliness in the elderly in various European countries ranges from 3 to 34% [7]. In Tomstad et al.’s study on loneliness among older home-dwelling people about 12% of the participants reported often feeling lonely [9].

Increasing evidence demonstrates important links between loneliness and health in older persons at both the physical and mental levels [6, 8–12]. Experiencing feelings of loneliness is associated with various forms of physical morbidity and chronic illness [7] and health-related quality of life [11]. Psychological attributes associated with loneliness include depressive symptoms, poor self-management, and low life satisfaction [6, 7, 10]. Loneliness has also been identified as an indirect predictor of mortality in older people [6, 13]. As its prevalence is expected to increase due to the ageing population, loneliness in older persons is increasingly considered a serious public health problem [7, 13–15].

However, loneliness, which is a subjective experience, has not been well-defined, as seen in the various definitions of loneliness found in the literature [7, 15]. Loneliness is most often defined as a negative, distressing or unpleasant experience regarding the quality or quantity of a person’s social relationships [7–10]. Many researchers explicitly focus on the social aspects of the concept and define loneliness as a discrepancy between one's desired and achieved level of social contact [8]. Although sometimes considered synonymous, loneliness and social isolation are distinct concepts. Social isolation has been defined as an objective state of having minimal social contact with other persons, while loneliness reflects a subjective state of perceived lack of desired affection and closeness with a significant other [7, 8]. This means that a person can feel lonely without being socially isolated or, conversely, be socially isolated without experiencing loneliness [16].

The absence of an acceptable social network is described as social loneliness [9]. When referring to a lack of meaningful relationships, regardless of the size of one’s social network, the emotional component of loneliness is stressed [9]. Loneliness also occurs on an existential level, where a reflection on the meaning of human existence arises [16]. This type of loneliness can be accompanied by feelings of intolerable emptiness, sadness and longing, and being aware of one’s own fundamental aloneness as a human being [17]. Research on loneliness from the perspective of older persons revealed that these aspects are interconnected, suggesting its complexity and multidimensionality [9, 12, 14]. Moreover, research has shown that older people perceive loneliness as a private matter—a feeling they would rather hide than talk about—which make research in this area particularly challenging [14]. Further clarification of this concept from the perspective of this population is needed to better understand what this phenomenon means for older persons and how they can be supported in dealing with it [15].

Multiple quantitative studies show that reduced mobility is a significant predictor of loneliness [6, 18, 19]. Community-dwelling older people with reduced mobility are challenged to deal with a multitude of losses. They report feelings of loneliness since reduced mobility limits their ability to leave the house, to participate in activities they formerly enjoyed, and to maintain ties with friends and family [3, 6]. Mobility limitations are thus considered a risk factor for the emergence and maintenance of feelings of loneliness. Notwithstanding, comprehension of the link between these two concepts is still absent and requires further exploration [3, 19]. Insights generated from the experiences of this at-risk population can help us to better understand loneliness and the underlying processes that lead to these experiences. Therefore, we aimed to investigate the experiences of loneliness in community-dwelling older persons with reduced mobility. By exploring the role of mobility in the occurrence or maintenance of loneliness more deeply, the underlying process of the experience of loneliness can be examined. Thus, we formulated our main research question: How do community-dwelling older persons with reduced mobility experience loneliness?

## Methods

### Design and setting

A qualitative study with a grounded theory approach [20] was performed to explore and theoretically explain older people’s experiences of loneliness and its underlying processes. The study population included Belgian community-dwelling older people with reduced mobility and experiences of loneliness. We collaborated with a home care organisation in Flanders, Belgium to recruit participants.

### Participants

Participants were purposively selected in collaboration with home healthcare providers of the White and Yellow Cross, the largest non-profit organisation for home nursing in Flanders, Belgium. The following inclusion criteria were used: (1) aged 75 years or older, (2) having reduced mobility, (3) experiencing feelings of loneliness, and (4) living at home and cognitively able to be interviewed. Patients were excluded if they had (1) an acute sickness causing mobility limitations, since the presence of this limitation can be temporary; (2) a chronic mobility limitation caused by an accident or a congenital disease, since ‘reduced mobility’ could be experienced in a different way and consequently have a different impact on the persons’ experiences of loneliness; (3) a physical disability; (4) a disease in a terminal stage, because of the direct influence this stage can have on mobility and the experience of existential loneliness; and (5) a diagnosis of depression (reported in the patient’s file), to avoid confusion between loneliness and a depressive disorder. The researchers contacted willing candidates to confirm their voluntary participation and to set a date for an in-depth interview. In addition to purposive sampling, participants were recruited using the snowball method in collaboration with earlier interviewed older persons and acquaintances.

The presence of feelings of loneliness was estimated based on the insight and expertise of the home healthcare providers. The possible presence of feelings of loneliness was sufficient to include the older person. Home healthcare providers could use the Jong Gierveld scale of loneliness [21], which was offered to help them recognise older persons experiencing feelings of loneliness. This scale considers loneliness as a personal experience and proposes 11 statements considered indicative of experiencing loneliness (e.g. experiencing a sense of emptiness, missing the company of others, having no one to talk to about day-to-day problems, etc.). Home healthcare providers used the scale by discussing with the older person which statements may be applicable to him or her. These data were passed on to the researchers.

Mobility limitations were classified using the scale of functional ambulation categories (FACs) [22], which breaks down the degree of a person’s mobility into six categories, where 0 stands for ‘non-functional ambulator’ and 5 denotes an ‘independent ambulator’. Older people were included in the study if they belonged to category 0 up to 4. This means that as soon as the older person can no longer walk independently or experiences problems with this (not being able to safely climb stairs, climb a slope, or walk on uneven surfaces), he or she was included. Older people who had no problems walking independently, belonging to category 5, were excluded from the study. The older person’s cognitive ability to conduct an interview was estimated by the clinical judgement of the home health nurse.

For participants recruited through the snowball method, the inclusion and exclusion criteria were evaluated by the researchers. If, after the interview was conducted, the participant’s cognition was judged to be inadequate, the researchers decided not to include the participant in the study. However, the presence of diagnosed depression could not be verified for these patients.

In this study, 30 older people were interested in participating after the first announcement about the research. After a second attempt at contact was made to inform them about the study, 8 older people did not want to participate because of personal or health-related reasons, and two of them were excluded because they had no problems with mobility (an FAC score of 5). Two older people were not interviewed because they were contacted after recruitment ended. In this way, 18 participants were interviewed. Three of the interviews were not used: two because the persons interviewed had limited cognitive function, as they could not answer the questions appropriately, and one because it became clear during the interview that the participant’s reduced mobility had been caused by an accident. Thus, a total of 15 interviews were included in the study.

### Data collection

Narrative data were collected from January 2018 until April 2018 using semi-structured in-depth interviews. The interviews were conducted at the participants’ homes. All participants were interviewed individually except for one couple, where both participants met the inclusion criteria and wanted to be interviewed simultaneously.

Before performing the interviews, we conducted a pilot interview to optimise the initial interview guide (see Additional file 1). Three researchers (MM, BH, SG) conducted all interviews and had no professional relationship with the participants. The length of the interviews ranged from 35 min to 1 h, 27 min. All interviews were digitally recorded and transcribed verbatim. For every interview, a descriptive and methodological report was made, and a first summary was written with the main messages of the interview [23].

All participants received oral and written information about the study and their rights as a participant. This information enabled the participants to give well-considered permission to participate in the research, which was confirmed by informed consent. The interview guide was refined throughout the study. It consisted of open-ended questions probing the experience and the underlying processes that make community-dwelling older persons with reduced mobility feel loneliness. The interview started by asking the respondents basic questions about their personal situation to make them feel at ease, obtain background data, and to better understand their story (e.g. questions about their former job, how many children they have, if they receive help in their environment, how they walk around the house, etc.). Next, the participants were asked to tell about their daily lives and activities (e.g. ‘Please describe to me what you did yesterday from the moment you got up until you went to sleep’). Using open and probing questions, the interviewers invited the older persons to describe their experiences in more detail, including pleasant moments and meaningful relationships. Special attention was given to their experience of ageing, reduced mobility, and loneliness.

### Ethical approval

The Ethics Committee of the Faculty of Medicine of KU Leuven approved the study (mp001699).

All methods were performed in accordance with the Declaration of Helsinki. Prior to the interviews, the participants were provided with written and verbal information regarding the study, its aim and the implications of participation. All participants gave their informed consent to participate and to use their anonymised data for publication purposes.

Particular attention was given to supporting the older person both during and after the interview. Time was done to clarify expectations with regard to the participant and to develop a relationship of trust with each participant. For many, it was an emotionally charged conversation during which the interviewer’s presence and genuine interest was appreciated.

### Data analysis

The process of data analysis was based on the comprehensive theory and practice-based Qualitative Analysis Guide of Leuven (QUAGOL) [23, 24], which is strongly inspired by the grounded theory approach.. The QUAGOL consists of two phases: preparation for the coding process and the actual coding process by means of a software program. The strengths of the guide lie in its underlying principles: a case-oriented approach with a continuous balancing of within-case and cross-case analysis, forward–backward movements using the constant comparative method and the combination of two analytical approaches (i.e. a holistic, creative approach and a classic thematic analysis). An extensive preparation of the coding process, instead of line-by-line coding, produces analytically and contextually meaningful ideas and codes, helping researchers to develop concepts and theoretical insights into the phenomenon. In accordance with the guide, a team approach was chosen that included 5 researchers in the analysis process (MM, BH, SG, BDdC, KDV).

The researchers started by reading and discussing the written interviews and reports on the team to capture the core ideas of each interview. A combination of within-case and cross-case analysis allowed us to elicit the uniqueness of each case while contextualising it in the broader context formed by the other cases. The common core ideas of all interviews were merged on a more abstract level and were used to develop a code list. Assigning meaningful text fragments to the codes and in-depth cross-case analysis of the codes was performed by means of the QSR NVivo11 software program.

Several strategies were applied to optimise the study’s methodological quality. Researcher triangulation and peer review were used on a regular basis to raise the credibility of the results. Peer debriefing with three external researchers with expertise in research and the care of community-dwelling older persons was conducted. The researchers applied bracketing on a regular basis to remain aware of their influence on, and presumptions about, the results. All of these strategies are incorporated into the systematic approach of QUAGOL [23, 24].

## Results

### Sample

The sample consisted of 15 older people (11 females and 4 men) between 75 and 92 years old. Most participants lived alone, of whom nine were widow(er)s and one was single. Five participants cohabitated with their partners. All but one (FAC 1) had a score of 3 or 4 on the FAC scale, which implies difficulties walking independently and/or needing supervision.

### Key results

All the participants expressed loneliness in their stories, but it appeared as an elusive, intangible phenomenon. It was difficult for the participants to indicate the presence of loneliness, as well as to describe what the phenomenon means. Although a few people explicitly expressed their loneliness during the interview, in most stories, loneliness was found to be implicitly present.

Older persons’ experiences of loneliness were described as an inherent part of the ageing process. Loneliness appeared to be linked to the constant, often simultaneous appearance of various age-related losses, among which reduced mobility played a significant role. Their experiences of loneliness were embedded in a story of loss, limitations, and meaninglessness, resulting in deep sadness. The participants felt like they were losing grip on their own possibilities and social connections. The confrontation with these limitations evoked a feeling of constantly reaching one’s limits without being able to address the situation. Not being able to think, act, and be like the person they used to be led to a feeling of meaninglessness. The interviewees experienced a lack of enjoyable activities, felt like they were a burden to others, and had the idea of being no longer of value to their environment. Eventually, they were left with a feeling of being unable to change the idea that the life they had once built up was slowly but inevitably slipping away.

Although experiences of loneliness were present in all interviews, there were differences in how they were expressed and the extent to which they dominated the participants’ stories. These differences can be partly understood through personal or contextual factors.

We used the case of Charles (fictitious name), one of the participants, to illustrate the profound experience of loneliness and the underlying dynamics of loneliness among community-dwelling older people with reduced mobility. This means that we broadened the perspective of Charles (a very rich interview in the scope of the research question) with the perspectives of other respondents to display the nuanced theoretical insights of this study in a more vivid, natural and dynamic way.

### The Case of Charles

Charles is 90 years old and lives with his wife in their home. They have been married for more than 60 years and have four children and four grandchildren who regularly come over for a visit and provide support where necessary. Charles moves around the house with a walking stick and uses a walker outside. A nurse comes by on a daily basis for hygienic care since Charles has osteoarthritis.

Charles tells us that the process of ageing is difficult and that he has increasingly fewer physical capabilities. He is no longer able to take care of the household properly, and he can no longer wash himself or put on his clothes. Basic movements become more difficult to perform, and he cannot change anything about them. He often depends on others to help him out. He speaks about this with tears in his eyes through which his grief becomes visible.

Charles continues talking about the loss of contact with peers who fall away one by one. He expresses the feeling of a loss of participation. When he goes to the monthly meetings of retired people, he does not feel at home there anymore. He is no longer able to just go and have a chat with everyone because he cannot move properly anymore. Therefore, he must sit still in his chair. In addition, Charles cannot sustain valuable encounters with others. The feeling of being left alone pops up on a more regular basis.

Another aspect is that Charles experiences that his own limits seem to come closer and closer. He continuously collides with the limits of his own abilities. Due to his reduced mobility, he also seems to be limited by his environment. Experiencing the death of peers confronts Charles with the approaching limits of his own life.

When we asked Charles if he feels lonely, he said the following with a shaky voice and tears in his eyes:

*I don’t truly feel lonely. I’m convinced that things are as they are, you can’t change it, you have to take life as it comes…and make the most out of it […] I don’t know how to explain it.*

### Loneliness as an elusive but deep human experience of sadness

Loneliness was expressed through all the stories of the participants, but as became clear through the story of Charles, openly communicating about their loneliness appeared to be a challenge for most of them. Only in a few interviews was loneliness more explicitly expressed when asked whether they were experiencing loneliness.

*I'm always alone on the weekends. From Friday afternoon to Monday afternoon. That is a long time. (...) Then, you think [sighs]: Why am I living here alone like this? Why am I still living here? Then, you say, gosh [already sighing]: Do I have to get up early again tomorrow, do I have to get up again tomorrow? (Alice)*

*Yes, sometimes I feel lonely. My daughter cannot stay with me all the time. Then, I see a couple walking outside and I think, ‘Here I am now’. Then, I am so…sad. (Elisabeth)*

It was difficult for most participants to both talk about the presence of loneliness and to describe what this phenomenon means. However, a thorough analysis of their stories and their underlying messages allowed us to uncover experiences of loneliness, their meaning, and what made them feel lonely. It became clear that loneliness appeared to be an inherent part of the ageing process accompanied by feelings of deep sadness. All of the stories were about loss, restrictions, and meaninglessness. It was precisely the occurrence and interaction of multiple experiences of loss, the associated feeling of being increasingly limited, and the negative emotions that accompanied these feelings that pointed to the complex, painful nature of loneliness among community-dwelling older people with limited mobility.

The feeling of deep sadness was found to be associated with feelings of grief, depression, and nostalgia in multiple interviews. Some older people expressed feelings of sadness, while others became emotional during the interview or reluctant to tell their story.

*(The experience of losing people close to me) is very painful. It truly makes me sad (becomes emotional). I often cry. When I’m alone. You’re sad that they’re gone, that you can’t hang out with them and can’t…it’s gone. (Lidia)*

Their feelings were often the result of an unfulfilled desire for deep social contact. The loss of meaningful interactions resulted in fewer and fewer people to fall back on, and eventually the feeling that they were on their own. Being or feeling unable to take the initiative to establish social contact due to functional and mobile limitations reinforced this fact. Feelings of sadness seem to be expressed mainly when they were alone in their home and on days with less social contact.Another expression of sadness manifested in the appearance of a generally negative and gloomy feeling. Older people who have these feelings experience less pleasure in activities, as a result of which they (temporarily) withdraw from social situations. Further, sadness can arise when one looks back on one’s life. Many participants thought back to whom they used to be, to what life was like, and to the people they lost. They told a story of grief and loss with regard to their ‘former life’ and described the time of the past as a better time.

*Because it was so much better in the early days, meaning more pleasant. The fact that you had many friends. In addition, now, there is no longer hope for that. When my friend lived here, she was always there for everyone and you could talk to her, you could tell her things. Now, nothing, now you have to process things alone. I miss that a lot. (Lea)*

### Loneliness as an inherent part of the ageing process

All the participants in this study struggled with a multitude of losses, many of which were related to the process of ageing. Ageing itself was seen as a gradual, inevitable process of decline, accompanied by continuous feelings of loss, particularly at the physical, social and existential levels. The constant and often simultaneous appearances of these experiences of loss were clearly linked to the participants’ feelings of loneliness. It was mainly the interactions between these losses that left the participants with a sense of meaningless and of living in a shrinking world.

#### The loss of functional capacities, mobility, and meaningful contact

The loss of functional capacities, mobility, and meaningful contact were the most prominent experiences in the older persons’ stories. When ageing, the participants lost their ability to perform certain activities, to leave their environment, and thus the possibilities of maintaining meaningful contact. All stories pointed more or less to the loss of a meaningful completion of the day with pleasant activities and encounters with others.

In many stories, the perception of loss of functionality and mobility went along with the feeling that they were no longer able to do anything.

*Ageing means…that you can’t do things the way you did them when you were younger. I danced all my life, my shoes were worn out. In addition, my husband too […] Yes, but now I’m stuck at home, because that is no longer possible. I can’t dance anymore. There are many things you have to say goodbye to when you become older. (Annie)*

Daily household, leisure, and social activities that used to be fun were abandoned. These activities became too difficult to perform for older people because their body was no longer able to provide the necessary strength and flexibility. Alternatively, they were avoided due to the risk of physical harm.

*Yes (I imagined growing older differently), actually yes, without the fall I would have had a different life. I would still be chatting around […] I truly hate this. This is the worst part of my life. Because you still want to. First, I truly enjoyed doing everything myself. My house, the cleaning and all of it. I was a happy person, I enjoyed everything […] Did my life change? Yes. (Lea)*

This experience is accompanied by an increasing feeling of dependence on others and the idea of having to stay at home and spend days that became monotonous and boring.

*Before noon, then I’m bound to my house. I have to wait until the nurses arrive. That is also very nice…it breaks the chain. (Charles)*

The loss of meaningful relationships was also expressed by the weakening of family ties, the loss or death of their loved ones, or the sickness of a spouse. The social contact that older people still have becomes more superficial, which means that in-depth contact was often absent. The weakening and the loss of family ties saddened older people and led to more regular feelings of being (left) alone. The fact that most of the participants indicated they had a great need for meaningful contact, and at the same time experienced a lack of it, created further isolation and an increase in feelings of loneliness. Many participants in this study were confronted with the death or loss of loved ones. The passing away of a spouse or child was described as a great, everlasting sadness that they carried with them for the rest of their lives.

All these experiences made the participants no longer appeal to meaningful people in their lives. As a result, several interviewees no longer took part in social activities, which in turn led to a further loss of social contact.

#### Living in a shrinking world

The constant struggle to deal with a multitude of losses brought out a feeling of living in a shrinking world. The experience of constantly reaching one’s limits without being able to address the situation was perceived in all stories. The participants saw their capabilities gradually reduced, which in turn gave them the feeling of living in an even smaller world. These feelings of limitation mainly manifested at the physical, social and existential levels and differed across participants.

On a physical level, the participants became aware that their loss of functional capacities was irreversible. They had to continue living with their physical limitations and were ‘stuck’ in their less functional body. In this way, they felt continuously confronted with the fact that their active life was over. Participants who were unable to leave their homes clearly sensed the boundaries of their environment.

*You’re stuck. That back, those arms […] There are also people, mobile enough, but the setbacks they’ve faced in their lives make them also struggle […] They say ‘We have that, we have to face that, but we still have to go on’. (Jack)*

The loss of meaningful social contact made the participants no longer appeal to meaningful people in their lives. They lost important sources of support, friendship, and pleasure in the activities they used to do together. Their social environment was gradually diminishing, leaving them isolated in their literally and figuratively shrinking world.

*That was truly the 4 of us [shows close bond with hands]. After one year or 2 she got cancer. We did everything the same. She was alone, I was alone. We travelled together and still did, yeah […] And then she got…She truly suffered. In addition, then she passes too. However, that is…That is truly something. Then, you are all alone [tears]. All alone. (Constantine)*

When the participants felt limited on an existential level, this was especially noticeable when they were confronted with the deaths of their peers. At these moments, the participants became aware of their own lives possibly ending, and they wondered when it would be their turn.

*That is truly bad. That touches you more than back in the days. Then, it was ‘a thing in the far future’. In addition, now, that’s close by […] You just feel that it is no longer possible. (Charles)*

The idea of further deterioration and once again being left alone were prominent in these experiences. The feeling of existential limitation was reinforced by feelings of meaninglessness.

### A sense of meaninglessness

A sense of meaninglessness was also present in almost every story. The participants indicated that they experienced a lack of enjoyable activities and meaningful and beautiful or pleasant moments. Experiences of meaninglessness were strongly connected with a feeling of a lack of self-worth. Their identity had been compromised; they no longer associated themselves with the person they used to be. This made them feel worthless. They felt that they were no longer of value to their environment or to society, and that they were a burden to the people around them.

*[What are the most beautiful and best moments currently?]*

*[silence] They don’t happen that often, in real life. Yes, when there is a visit, when the children come by. However, that doesn’t happen that often […] Back in the days we more often went to them. Yes, we visited them more often than they visited us, because they are working and we weren’t anymore, so we could move more freely. […] Much is changed you know [emotional]. (Robert)*

The sense of being a person who has meaning for and is appreciated by others, through the performance of meaningful activities, was lost and replaced by the feeling of becoming increasingly dependent on help and support from others. Many participants indicated that they avoided certain social events because they did not want to be a burden to others, and because they did not want to be confronted with their loss of functionality in the eyes of others.

*In the past, I always used the bus to go and visit things, but now I don’t do that anymore. I don’t want to be a burden to others, because I’m not that mobile anymore. So I stay at home. […] Those people, they go sometimes. They have to hold them, help them. I don’t want to be a burden to someone else... (Mary)*

Many of the interviewees described how they filled in their time with activities that only served as a hobby. Some people even asked themselves why they were still alive, what they were still doing here, and what meaning they still had in their life and in the lives of others.

*Yes, then I think, ‘Why do I live alone like this? Why do I even live’? Then you say, ‘Well, do I have to get up in the morning, tomorrow, do I have to again…that you don’t anymore...it’s like...but you don’t want to die and I thought like, ‘God, dying, that must be terrifying’? (Lidia)*

It was striking that although some older people still had much social contact, they experienced a lack of in-depth contact. This trend of superficiality was caused by an increasing number of encounters with others that were based on necessities such as providing transport or helping with domestic tasks.

*At night, the nurse still comes around, but that is for 10 minutes. Putting my shoes out, or my stockings, or putting on my pyjamas, hanging up my clothes, … […] However, that doesn’t take too long, right…*

*[And what is very important in your life?]*

*Pff. Yes, that more people come to visit me. That more people…If only for one hour […] It’s enough that someone visits me from time to time. Yes, that you can talk to someone and ask how it is and this and that. (Alice)*

The participants also experienced a loss of community because they have lost many people from their generation, and they no longer feel at home in today’s society. Examples that were discussed were the individualisation of society, which promotes the loss of spontaneous chats and help from neighbours, and the reduction of time and in-depth contact that children (can) give to their parents. These experiences made the participants feel that they have been left alone and live in a neighbourhood that they no longer seem to be a part of.

*Do you know what is sad currently? People are no longer truly in touch with each other. The people here…I have lived here a long time, in this house. In addition, when I moved in here, that was here, the people, they talked to each other on the street at night on a bench and we laughed. However, because I was the youngest at home, I was also the youngest here. They have all passed away. Now it is even too much to say hello. People. That is horrifying. (Lea)*

In some stories, the meaning of life was explicitly questioned, mostly when valuable activities and contact with meaningful persons disappeared in the life of the older person.

*Then, I set the alarm, that I don’t fall asleep for sure and that I’m awake in time. Then, you get up. However, yes, what is the purpose of your day? I’m telling you, the nurse comes and by noon they bring my lunch. Is that a purpose to you? I don’t have a purpose or goal. Goal? No, you’re alive, you’re alive. That’s truly hard for me. You get up and you have to try to make the most of it. However, in the end, what is the goal? I don’t have a purpose anymore in my life. No, no, that is over. The only goal I have is that I still have my son [cries]. (Leo)*

### Influencing factors

Although the experience of loneliness was present in all the stories, the analysis revealed differences among the interviewees. Although no saturation was achieved for a good understanding of the underlying factors, the study revealed many examples suggesting the role of personal and contextual factors in reducing or enhancing older persons’ experiences of loneliness.

#### Contextual factors

Contextual factors such as one’s social network, and the course of life and major events in the participants’ lives, appear to somehow influence their experiences of loneliness. The presence of one or more persons that the older person can always turn to and from whom they receive help and care positively impacts their experiences. The company of these meaningful instances of contact and the enjoyable moments spent with these people were described as very important in the participant’s life.

*My bed has already suffered, my furniture too. However, yes, I can bear that, you have to bear that. I like that. Among grandchildren, you feel alive again. They even give you more joy than your own children. (Maria)*

Participants with fewer social contacts indicated that they missed being able to chat with someone. Being physically alone seemed to aggravate the emergence of negative feelings that may lead to loneliness. A few older people who explicitly reported that they felt lonely experienced their negative feelings mainly on days when they had no social contact or company. These days were described as difficult and lengthy.

How the participants had lived in the past, what they were used to at the time, or what choices they made at the time helped us to understand how they experience loneliness today. For example, loneliness was felt differently by an older person who had no children compared to an older person who had raised a family. Some participants reported that they never had friends in the past and therefore did not miss these contacts now. Older people who never used to go to the city did not miss these activities.

*I’m telling you, I’m used to being alone, I’ve been alone whole my life. That helps a lot. It’s not like you suddenly have to face things alone. (Blanche)*

Some major life events reinforced the participants’ feelings of loneliness through the heavy impact on their life and the irreversibility of these events. For example, the death of a spouse or child was said to be a major loss for the older person. Some participants said they lost ‘their everything’, they felt stuck, and no longer saw the possibility of getting out of their situation.

Another noteworthy example refers to weather conditions. According to some interviewees, good weather positively impacts their mood and courage; they can go for a walk or sit outside with their neighbours and enjoy good weather. In contrast, when the weather is grey, dark and cold, as in winter, some participants explained how they experience the days as too long, resulting in stronger negative feelings.

#### Personal factors

Differences in the experience of loneliness can also be understood through personal factors. The participants’ stories revealed differences in the ways they spend their day and experience daily activities. We observed differences in their personal drive, which can be more positive or negative. For example, some participants described how they can enjoy activities (such as painting) or can find pleasure in day-to-day activities such as household tasks. A few actively search for meaning in their lives; they still want to mean something to others and thus to add value to their lives. Doing someone a favour makes them still feel important in the lives of others.

*[Regarding one] of my sons-in-law, well, the granddaughter comes with her boyfriend. A pair of pruning shears was broken. It was truly broken. He comes here and the fact that I can fix that, that was truly fun. I can recommend that. It makes you look forward to the next thing. (Charles)*

Other participants lacked this kind of positive drive and just tried to find activities to fill the void of time and change their mind. These older people explained how important it was to stay busy when feelings of loneliness were present, as this was the only way to ‘keep going’.

*[What are you doing now?]*

*Yes, actually, not much anymore. Yes, like this cross word puzzle. You have to do something.*

*[Is it correct that you are looking for things to do?]*

*Yes, yes, I look, yes, yes. Sometimes I watch TV, yes that is good. Then, an hour has soon passed. (Jack)*

Another example referred to how older persons deal with the process of ageing. A few participants seemed to experience it in a more positive way. They said, for example, that they had learned to live with the fact that some things are not going as well as they used to. They have tried to move forward in life and retain as much independence as possible. At certain moments during the interview, they demonstrated a fighting spirit, an attitude to not sit back and complain. They were grateful for the fact that they were able to grow old.

For most participants, however, getting older was seen as a challenge. They felt like they had to accept the situation as it was, accompanied by negative feelings. The interviewees realised that they could not do anything about it and that it was pointless to protest. They knew they had to live with the situation as it was, but had a hard time with it.

## Discussion

The aim of the study was to better understand loneliness as experienced by community-dwelling older persons with reduced mobility. Loneliness was experienced through all the stories of the participants, but it became clear that it is a complex and difficult to grasp phenomenon. Loneliness is experienced as an inherent part of the ageing process characterised by losses, limitations, and meaninglessness. The participants described how they have experienced losing their grip on the world in which they live, and how they feel isolated in a literally and figuratively shrinking world. These results are in line with the findings of Taube et al.’s study [4], which described loneliness as ‘being in a bubble’, excluded from the ongoing world because of many losses. In the same vein, Sjöberg et al. [25] interpreted older persons’ experiences of loneliness as ‘being disconnected from life’, pointing to the feeling of abandonment and living a meaningless life.

Each interview revealed a story of losses. The participants talked about kinds of losses, all related to the ageing process. They elaborated extensively on their loss of functional capacities and mobility, and illustrated how these losses contributed to their feeling of living in a shrinking world and their sense of meaningless. It is mainly the interactions between these losses that places older persons at risk for loneliness, as also evidenced in the literature [3, 4, 25, 26]. Sjöberg et al.’s [25] participants described feeling trapped in a frail, deteriorating body, limiting their access to the world (‘life had come to a standstill’), resulting in increased isolation. This feeling of isolation, along with the loss of close ties and meaningful engagement, causes deep feelings of loneliness according to older persons [3, 4, 25, 26].

Our findings revealed that it is precisely older persons’ feeling of inability to address the situation that results in deep sadness and the sense of no longer being of value to their environment. These feelings of sadness and emptiness can lead to a deep sense of hopelessness, as reported by Taube et al. [4]. The participants in their study described how they felt invisible, not noticed, or understood. Facing this indifference strengthens the experience of being worthless [25]. Sjöberg et al. [25] related these feelings of sadness, emptiness and abandonment to existence in a ‘vacuum’, hindering older persons from sharing their lives, thoughts and experiences with others. This finding is of concern as older people living alone is a growing group in many countries, particular in Europe where more than 30% of all persons 65 + live alone [27]. The Covid-19 pandemic has also painfully exposed the vulnerability of this group of older persons.’ [28, 29].

Loneliness was expressed in all participants’ stories, but speaking about loneliness was difficult for most of them. A constant state of loneliness among older persons has also been shown by Taube et al. [4]. Living in a bubble is not optional, as described in their study [4]. However, many participants were reluctant to express their loneliness during the interview or were not able to describe what this phenomenon means to them. The difficulty of speaking about experiences of loneliness has been discussed by many researchers [4, 12, 30, 31]. This finding might be related to the stigma associated not only with loneliness, but also with ageing and frailty [12]. For many older people, loneliness may be a private matter and thus hard to talk about with professionals [12, 30]. This finding, in combination with the elusiveness and intangibility of this phenomenon, suggests a more profound experience of loneliness than at first might appear in the participants’ stories, which is an inherent part of the older person’s life. This may explain why this phenomenon has often been overlooked and underestimated by caregivers [32].

Although the experience of loneliness was present in all stories, the analysis revealed differences within and among individuals. The experience of loneliness can be more or less present, or expressed as more or less disturbing.

The differences in experiences between participants can be partly understood through personal and contextual factors, as has also been suggested in previous research. Sociodemographic variables and personality, health-related factors, social support and activities, and the characteristics of the living environment have been identified as potential influencing factors [6, 33, 34]. Loss of mobility had a prominent place in the participants’ experiences of loneliness and clearly contributed to these experiences. As confirmed by Smith [3, 26], reduced mobility interferes with the participants’ ability to connect with others and to socially engage. However, it was not possible to fully understand the role of reduced mobility in the occurrence or maintenance of loneliness, as the phenomenon is a complex interplay of age-related losses and personal and contextual factors. More research is needed to better grasp the underlying processes and dynamics that may mitigate or reinforce experiences of loneliness.

In contrast to our findings, Taube et al. [4] pointed to the positive coexistence dimension of loneliness, offering older persons time to reflect, the freedom to make decisions, and opportunities to create meaningfulness. In our study, we only observed in some interviews that the older persons focused strongly on positive things and how they tried to deal with loneliness from a positive perspective as a way to illustrate and maintain their self-esteem. This may refer to the older person’s personality or strategy to cope with the stigma related to loneliness and ageing [3, 12, 26, 31]. The purposeful sampling of older people with reduced mobility might also explain why we did not detect this positive dimension of loneliness in our sample.

Research on loneliness, from the perspective of community-dwelling older persons, is scarce, but all available evidence supports the idea that older persons are at risk of loneliness not so much due to a lack of activities, social networks, or life energy, but rather due to their search for meaningful interactions, in-depth social contact, and fewer restrictions [32]. The importance of these findings is underlined by comparison with other perspectives on older persons’ loneliness. Larson et al. [32] revealed a remarkable contrast between older persons’ experiences and perceptions of significant others. In contrast to the older persons themselves, significant others (such as family and friends) related experiences of loneliness primarily to a lack of activities, not taking part in social events, and the loss of a fighting spirit [32]. It is clear that these differences in perceptions may create tensions and prevent older persons from receiving the kind of care or support they need to age healthily.

One limitation of this study has to do with the difficult recruitment of participants who met the criteria and were willing to share their experiences. Purposive sampling was complemented with snowball sampling, thereby limiting the analytical generalisability of our findings. Although we could not use theoretical sampling, we obtained a relevant variability of characteristics in our sample.

The choice to recruit through caregivers is not an optimal option, but allowed us to recruit participants who expressed interest in our study and were willing to participate. We considered the experiences and expertise of caregivers in the care of older persons as a good basis for discovering older patients with feelings of loneliness. The provision of the De Jong Gierveld scale was primarily intended to encourage caregivers to look beyond "visibly socially isolated patients”. This recruitment method resulted in participants willing and able to share relevant and rich data in response to our research question. However, whether this method also resulted in the most optimal sample to describe the phenomenon of loneliness in older community-dwelling older persons remains a question. The selection of participants through referrals from earlier participants (snowball method) could possibly have reinforced some selection bias.

The delicate nature of the phenomenon under investigation together with the vulnerability associated with aging formed another challenge in this study: to bring up a phenomenon that people may prefer not to talk about. Recruitment through caregivers or acquaintances (with whom they had a relationship of trust) led us to participants who showed an interest in participating in the study. Subsequently, much time and care was taken to provide the participants with all the necessary information, assurances, and reassurance to make their choice. The focus of the interviews was placed on daily life with its beautiful and more difficult moments. All the interviews led to an open conversation in which beautiful experiences alternated with more difficult or painful emotions. Ample time was always taken after the interviews to talk with the older person so that the interviewer could uncover any potentially painful or troubling feelings or thoughts and provide the necessary support*.*

Using the QUAGOL and a mix of strategies to optimise the methodological quality (triangulation, peer debriefing, peer review, member check), saturation was achieved only at the level of the core concepts. The main messages could be clearly described, but the underlying dimensions, variations and relationships could not be sufficiently elaborated. Although loss of mobility made up a prominent place in the participants’ stories and clearly contributed to their experiences of loneliness, it was not possible to fully understand the role of reduced mobility in the occurrence or maintenance of loneliness. More research is needed to better understand the underlying processes and dynamics that may reduce or reinforce experiences of loneliness.

The interviewers’ lack of experience before the start of this study may have affected the data collection. The constant use of peer review and the presence of expert qualitative researchers on the team were helpful to obtain rich and nuanced data.

## Conclusions

Loneliness is a complex and difficult phenomenon to understand and describe. Listening to the perspective of home-dwelling older persons helps us see that this is a deeply human, painful experience that is an integral part of aging. Loneliness is more than simply ‘being alone’. It involves the loss of different parts of the life of the older person, often leaving them with the feeling of losing grip on the world in which they live and of no longer being of value to their environment. Exactly this feeling of being abandoned and no longer having a meaningful life makes this group of older people very vulnerable. The difficulty to speaking out these profound feelings of sadness involves the risk to underestimate the problem of loneliness among home-dwelling older persons with reduced mobility. How to adequately assess and support this vulnerable group should thus be given priority in health care. This explorative study to understand loneliness from the older person’s perspective is a crucial start for a better understanding of how to recognise and address experiences of loneliness in older persons. Being aware of this vulnerability, health care professionals can find how to support older persons in their search for meaningful interactions. They are in a privileged position to detect persons at risk of loneliness and to help them add meaning to their life. This requires a dignity supported approach, competence to assess the complexity of these older person’s life experiences and to identify or even anticipate feelings of loneliness.

Further research is necessary to refine and strengthen the results, and to further explore the role of reduced mobility in the experience of loneliness.

## Supplementary Information

**Additional file 1. **Interview Guide.

## Data Availability

The datasets generated and/or analysed during the current study are not publicly available due to privacy concerns but are available from the corresponding author upon reasonable request.

## Abbreviations

MM: Marika Moeyersons
BH: Brooke Huyghe
SG: Sacha De Groof
BDdC: Bernadette Dierckx de Casterlé
KV: Kristel De Vliegher
KM: Koen Milisen
