# Supplementary material for: ‘Living in a shrinking world’—The experience of loneliness among community-dwelling older people with reduced mobility: a qualitative grounded theory approach
Source: BMC Geriatr. 2022 Apr 6;22:285. doi: 10.1186/s12877-022-02998-5 (PMC8984073; doi:10.1186/s12877-022-02998-5)
Supplement: Supplementary file 1 — Additional file 1. Interview Guide. [file 12877_2022_2998_MOESM1_ESM.docx]

**INTERVIEW GUIDE**

INTRODUCTION

Information about the study and ethical and practical issues

WARM-UP QUESTIONS (to obtain some personal information on the participants and put them at ease; these data will further guide the interview)

- How old are you?

- What used to be your occupation? What have you studied?

- Are you married?

- Do you have any children?

- Do you live here at home alone?

- Who do you receive help from in your surroundings?

- How do you move around the house (bedroom, stairs)? Do you still use the upper floor or basement? Where do you sleep? Do you use any assistive devices?

- If you need to go somewhere, how do you do it? Where do you go most of the time? Is it frequent?

OPENING QUESTION

- Could you describe a day from when you get up to when you go to sleep? (Does every day look like this to you? Is it like this every day?)

IMPORTANT THEMES (selection of themes/questions according to the participant’s personal context and the course of the interview)

- Positive experiences

- What do you enjoy doing? (What makes you so enthusiastic about this/love doing this?)
- During your day/week, what are your best or most enjoyable moments?
- What is truly important to you in your life? Why is this so important for you?

- Meaningful relationships

- Who are the most important people to you in your life?
- Who comes to visit you? (How often do you see these people? When did you last see these people?)
- Who can you rely on at difficult moments? What does this person do for you in difficult moments?

- Less pleasant experiences

- What do you like less about your life?
- Can you tell us about a day that was less fun? What happened on that day? How do you cope with it?
- Can you give an example of a day that goes by less quickly?

- Mobility

- Can you tell us about the time/period that you started to have difficulties moving around?
- Are there things you no longer do because of your reduced mobility?
- What has changed in your daily life/contact with others/hobby/activities? In what way has your life changed due to your reduced mobility?
- Have you ever fallen? How did this affect your life?

- Loneliness

- Could you give a score out of ten on how well you feel most of the time? (using a scale, ‘I feel good’ (a score of 0) and ‘I do not feel good at all’ (a score of 10)
  - Do you always feel this way? What makes you not always feel well/always feel good?
  - Are there times when you feel better/worse (indicate on the scale)?
  - What does it mean to you to ‘feel good’?
- Are there times when you feel lonely? Could you describe a moment when you feel lonely?
  - What do you think then? What do you do? What would you like to do at those moments? What do you feel then? How do you cope?
  - Do you sometimes have bad days, days that you feel down, depressed or desperate?
  - What makes you not feel lonely?

- Growing older

- Please tell us what ‘getting older’ means to you. Could you tell us in a few words what it means to you to be getting older? What does it mean to you to be ___ years old?

CLOSING QUESTIONS

- If you had to explain in a few words to your doctor what it means to you to be lonely/older, what would you say?
- If you could give one tip to support people when they are feeling lonely, what would it be?
- Is there anything else you would like to add to this interview?
- How did you experience this interview?
- Were there things you had a hard time telling?

MORE IN-DEPTH QUESTIONS (inviting us to provide more information or clarification, or to go deeper into the subject matter)

- Can you tell me slightly more about that?
- How did it make you feel/how did you experience it?
- How did it come about that...?
- What was going through your mind at the time?
- What happened then?
- Since when have you experienced this? Has it always been like this? Was it different before?
- What impact did this have on your life/daily life?
- Is that something that happens often?
- What do you mean by that?
- What makes you do that?
- Why do you/why do you not…?
- What does it mean to you?
- What makes you so excited about this?
- What exactly is difficult?
